# Free to be Healthy? Free Will Beliefs are Positively Associated With Health Behavior

**DOI:** 10.1177/00332941241260264

**Published:** 2024-06-08

**Authors:** Tom St Quinton, A. William Crescioni

**Affiliations:** School of Humanities and Social Sciences, 4467 Leeds Beckett University, Leeds, UK; Department of Psychological Sciences, 6177 Texas Tech University, Lubbock, TX, USA

**Keywords:** Free will beliefs, health behavior, theory of planned behavior, prosocial behavior, beliefs

## Abstract

Previous research has demonstrated that a stronger belief in free will contributes to a variety of socially desirable behaviors. We assessed the correlation between free will beliefs and health behaviors. Four studies (*N* = 1172) provide evidence that belief in free will is positively associated with health protective behaviors (e.g., physical activity, fruit and vegetable consumption, low fat diet) and negatively associated with health risk behaviors (e.g., alcohol consumption, smoking, unhealthy snacking). In relation to the respective health protective and health risk behaviors, we found free will beliefs were more strongly correlated with physical activity and alcohol consumption, respectively. We also found free will beliefs were associated with key social cognition determinants (e.g., attitude, subjective norm, perceived behavioral control, and intention). Overall, our results suggest that belief in free will can have important consequences for health behavior. This contributes to current theorizing about the implications of believing in free will.

## Introduction

### Belief in Free Will

The extent to which people have free will is a long running and controversial philosophical debate. Philosophers have long debated not only whether free will exists, but what the concept of “free will” even means. Definitions of free will within philosophy encompass a wide range of positions, from the belief that free will entails a negation of determinism (libertarian free will) to the idea that free will is fully compatible with determinism (compatibilism; Van Inwagen, 2008). Within psychological science, the belief in free will is typically defined as the belief that individuals are able to exert at least some agentic control over at least some of their behaviors (Baumeister, 2008). Rather than assessing this belief dichotomously, most psychological scientists assess the belief in free will along a scale from relatively little belief in free will to a relatively greater belief in free will. As well as measuring existing free will beliefs, researchers have also devised methods to manipulate the extent to which individuals believe in free will. Although such manipulations do not generally produce dramatic shifts in free will belief, the small changes they do induce have been linked to significant behavioral changes as well (see Genschow et al., 2023 for a review). Experimentally reducing belief in free will has been shown to produce increased cheating (Vohs & Schooler, 2008), greater aggressiveness (Baumeister et al., 2009), and more prejudice (Zhao et al., 2014). Moreover, believing less strongly in free will reduces perceived autonomy (Alquist et al., 2013) and counterfactual thinking (Alquist et al., 2015), impairs self-control (Rigoni et al., 2012), and leads people to set less meaningful life goals (Crescioni et al., 2016; Zhao et al., 2023). Correlational evidence has also found that greater free will belief is associated with greater perseverance (Li et al., 2018) and self-efficacy (Crescioni et al., 2016), and reduced conformity (Alquist et al., 2013; Moynihan et al., 2019).

### Belief in Free Will and Health Behavior

Most research on belief in free will has examined prosocial behavior. However, there is some evidence that free will beliefs can have a role to play in health risk behaviors. Vonasch et al. (2017) found people with weaker belief in free will had a greater history of alcohol consumption and smoking, and were less successful at quit attempts. A similar trend was found for smoking. Weaker free will beliefs have also been associated with greater gambling behavior (St Quinton et al., 2022). The relationship between belief in free will and risky health behaviors could be a consequence of self-control and motivation. That is, people harboring weaker belief in free will may lack the requisite motivation to exercise the self-control needed to abstain from such behavior (Baumeister et al., 2008). They may also be less motivated to overcome urges and impulses (Baumeister et al., 2009). After all, if a person believes that their behaviors are predetermined, that person may see little value in attempting to exercise control over their own behaviors.

The extent to which a person believes in free will could also play a role in health protective behaviors. Free will beliefs are characterized by agency and choice, both of which are required to successfully manage health. Moreover, health protective behaviors such as physical activity and fruit and vegetable consumption also require perseverance, self-efficacy, self-control, and autonomy (Crescioni et al., 2011; Ntoumanis et al., 2021). Given that research has demonstrated a relationship between these traits and free will beliefs (Alquist et al., 2013; Crescioni et al., 2016; Li et al., 2018; Rigoni et al., 2012), the latter could therefore be associated with health protective behaviors. For instance, someone who does not believe they possess free will may feel as though their own health is ultimately not under their control. This person might well reason, then, that efforts to exercise, eat well, or abstain from alcohol would be pointless, as the future (including their own future behaviors) has already been predetermined.

### Belief in Free Will and the Theory of Planned Behavior

Health behavior can be explained using theories of social cognition, such as the theory of planned behavior (TPB; Ajzen, 1991). The TPB suggests behavior is driven by intention, which represents a person’s willingness to participate in the behavior. Intention is influenced by attitude, subjective norm, and perceived behavioral control (PBC). Attitude represents a person’s evaluation of the behavior; subjective norm concerns the perceived social approval to engage in the behavior; and PBC relates to a person’s perceived ability to undertake the behavior. Research applying the theory has found model constructs explain significant variance in intention and behavior (e.g., Armitage & Conner, 2001; Cooke & French, 2008; McEachan et al., 2011; Steinmetz et al., 2016).

In addition to theory constructs, the TPB provides room for additional individual and social influences on behavior. Specifically, these additional influences, referred to as background factors, are theorized to operate through model constructs (Ajzen, 2011). For example, research has found theory constructs to mediate the effects of personality (Conner & Abraham, 2001; McEachan et al., 2010) and empathy (De Leeuw et al., 2015) on health behavior. Pertinent to the present study, St Quinton et al. (2022) showed that greater belief in free will is positively associated with both PBC and attitude, and Hagger and Hamilton (2022) recently found the effects of free will beliefs on intention to engage in vaccine boosters was fully mediated by attitude, subjective norm, and PBC. It could therefore be that free will beliefs affect health intentions and behaviors by operating through TPB constructs.

### Overview of Studies

The present paper presents the results of four studies testing the relationship between free will beliefs and several important health behaviors. Specifically, we tested the hypothesis that believing more strongly in free will would be associated with an increased likelihood of engaging in health protective behavior and a decreased likelihood of engaging in health risk behavior. Study 1 first tested the association between free will beliefs and past health risk and health protective behaviors. These relationships were then explored in prospective measures of health behavior in Study 2. Study 3 further examined these associations whilst controlling for constructs conceptually related with human agency. Finally, Study 4 examined the relationship between free will beliefs and health behavior whilst considering the role of TPB constructs.

## Study 1

### Purpose

Study 1 aimed to replicate and extend previous work by establishing a relationship between free will beliefs and health risk and health protective behaviors. We hypothesized that belief in free will would be positively associated with health protective behaviors and negatively associated with health risk behaviors.

### Method

#### Procedure

A cross-sectional design was used. Participants were eligible if they were aged 18 years or older and spoke English. Participants were recruited through Prolific, an online crowdsourcing platform for participant recruitment. Once accessing the website, participants read detailed study information and provided consent to participate. Participants then completed the questionnaire assessing demographic details, participation in past health behavior, and free will beliefs. Once complete, participants were thanked for their participation and debriefed. The sample size for all four studies was based on the recommendations of Schönbrodt and Perugini (2013) who indicate 250 participants are needed to achieve stable small to moderate correlations. All studies presented received ethical approval from the University Faculty ethics committee.

#### Measures

##### Demographics

Measures were taken of age and gender.

##### Health Behavior

Participants were asked to report their engagement in six important health behaviors: fruit and vegetable consumption (consuming five portions of fruit and vegetable per day); physical activity (undertaking at least 30 minutes of moderate physical activity five or more times per week); low-fat diet (consuming a low-fat diet); alcohol consumption (drinking more than the recommended weekly units); unhealthy snacking (consuming unhealthy snacks); and smoking cigarettes (the extent to which they smoked cigarettes). Participants were asked about the extent to which they engaged in these behaviors during the previous four weeks. All behaviors were measured using a single item on a seven-point scale ranging from 1 (never) to 7 (always) (e.g., In the past four weeks, I have eaten fruit and vegetables). Single items were used to measure behavior to limit participant burden. This has been applied elsewhere in studies assessing multiple health behaviors (e.g., Conner et al., 2022; Schüz et al., 2021; Sheeran & Conner, 2019). To ensure participants understood behavioral definitions, descriptions of each behavior preceded measures. Higher scores represented greater behavior for all behaviors.

##### Free Will Beliefs

We assessed free will beliefs using the Free Will Inventory (FWI; Nadelhoffer et al., 2014). The FWI comprises subscales of five items each measuring belief in free will (e.g., “People always have the ability to do otherwise”, *α* = .72), determinism (e.g., “Everything that has ever happened had to happen precisely as it did, given what happened before”, *α* = .78), and dualism (e.g., “The human mind cannot simply be reduced to the brain”, *α* = .66). All items adopted 7-point rating scales ranging from 1 (strongly disagree) to 7 (strongly agree).

#### Analyses

We conducted a correlational analysis testing the bivariate correlation between each of the six health measures and the three FWI subscales.

### Results

A total of 297 participants took part in the study (174 males, 121 females, 2 not reported; *M*_*age*_ = 22.51, *SD* = 2.85). As shown in Table 1, the free will subscale was significantly positively correlated with physical activity (*r* = .625, *p* = < .001), fruit and vegetable consumption (*r* = .382, *p* = < .001), and low-fat diet (*r* = .477, *p* = < .001). Free will beliefs were significantly negatively correlated with alcohol consumption (*r* = −.307, *p* = < .001) and smoking (*r* = −.289, *p* = < .001). There was no correlation between free will beliefs and snacking behavior (*r* = −.080, *p* = .171). Finally, there were no correlations between the health behaviors and belief in determinism (all *p*s > .05) and dualism (all *p*s > .05). Full results of correlational analysis are presented in Table 1.

**Table 1.** Study 1. Means, Standard Deviations, and Zero-Order Correlations (*N* = 297).

	M (SD)	1	2	3	4	5	6	7	8	9
1. Physical activity	4.13 (2.14)	—								
2. Fruit and vegetable	4.06 (2.02)	.471***	—							
3. Low-fat diet	4.02 (2.00)	.485***	.760***	—						
4. Alcohol consumption	3.85 (1.81)	−.236***	−.252***	−.291***	—					
5. Smoking	2.80 (2.16)	−.321***	−.299***	−.286***	.267***	—				
6. Unhealthy snacking	3.28 (2.11)	−.155**	−.248***	−.189**	.237***	.540***	—			
7. Free will	4.24 (1.87)	.625***	.382***	.477***	−.307***	−.289***	−.080	—		
8. Determinism	2.94 (1.36)	−.053	−.103	−.096	.060	.032	.083	−.045	—	
9. Dualism	4.06 (1.60)	.002	.021	−.042	.003	.078	.047	−.030	.094	—

*Note.* **p < .01, ***p < .001.

### Discussion

Study 1 established a relationship between free will belief and health behavior. Specifically, and as expected, there were significant positive relationships between free will beliefs and all health protective behaviors and significant negative relationships between free will beliefs and all health risk behaviors, save for unhealthy snacking. Using Cohen’s guidelines (Cohen, 1992), effect sizes for these relationships were large for physical activity; medium for fruit and vegetable consumption, low-fat diet, and alcohol consumption; and small for smoking. The positive relationships suggest greater free will beliefs are associated with behaviors that are positive for health, and the negative relationships suggest weaker beliefs in free will are associated with behaviors detrimental for health. Although these findings demonstrated a relationship between belief in free will belief and health behavior, a limitation of the study was the focus on past behavior. Study 2 attended to this issue by examining the relationship between free will beliefs and prospective health behavior.

## Study 2

### Purpose

The purpose of Study 2 was to replicate the findings of Study 1 using measures of prospective health behavior. Similar to Study 1, we predicted that belief in free will would be positively associated with health protective behaviors and negatively associated with health risk behaviors.

### Method

#### Procedure

A prospective correlational design was used. Specifically, after completing the first questionnaire, participants were contacted four weeks later to complete self-reported measures of the six health behaviors. The study used the same recruitment strategy to that adopted in Study 1.

#### Measures

##### Time 1

Measures were taken of age and gender. Like Study 1, the FWI (Nadelhoffer et al., 2014) was used to measure belief in free will, determinism, and dualism.

##### Time 2

The six health behaviors were measured using the same items adopted in Study 1.

#### Analyses

We again computed correlations between the FWI subscales and each of the six measures of the health behaviors. Following this, a series of hierarchical multiple regression analyses was undertaken to understand the predictive utility of free will beliefs on the health behaviors when controlling for belief in dualism and determinism. To do this, the health behavior was the dependent variable and belief in dualism and determinism the independent variables (Step 1). Free will beliefs were then entered at Step 2.

### Results

Data were collected on 262 participants at T1 (162 males, 100 females; *M*_*age*_ = 22.60, *SD* = 2.84) and 249 participants at T2 (155 males, 94 females; *M*_*age*_ = 22.65, *SD* = 2.84). There were no differences between those completing both assessments and those completing only T1 measures. Results showed similar correlational patterns to Study 1 (see Table 2). Specifically, the belief in free will subscale was significantly positively correlated with physical activity (*r* = .590, *p* = < .001), fruit and vegetable consumption (*r* = .412, *p* = < .001), and low-fat diet (*r* = .457, *p* = < .001). Moreover, free will beliefs were significantly negatively correlated with alcohol consumption (*r* = −.344, *p* = < .001), smoking (*r* = −.322, *p* = < .001) and, unlike Study 1, snacking behavior (*r* = −.146, *p* = .021). Like Study 1, there were no correlations between the health behaviors and belief in determinism (all *p*s > .05) and dualism (all *p*s > .05).

**Table 2.** Study 2. Means, Standard Deviations, and Zero-Order Correlations (*N* = 249).

	M (SD)	1	2	3	4	5	6	7	8	9
1. Physical activity	4.02 (2.06)	—								
2. Fruit and vegetable	4.03 (2.03)	.491***	—							
3. Low-fat diet	3.89 (2.02)	.485***	.771***	—						
4. Alcohol consumption	3.83 (1.82)	−.300***	−.248***	−.253***	—					
5. Smoking	2.72 (2.13)	−.321***	−.339***	−.288***	.280***	—				
6. Unhealthy snacking	3.20 (2.06)	−.205**	−.262***	−.219***	.219***	.547***	—			
7. Free will	4.19 (1.85)	.590***	.412***	.457***	−.344***	−.322***	−.146*	—		
8. Determinism	3.07 (1.39)	−.047	−.098	−.096	.040	.062	.117	.005	—	
9. Dualism	3.90 (1.59)	.026	−.018	−.065	−.017	.081	.055	−.021	.116	—

*Note.* *p < .05, **p < .01, ***p < .001.

Full results of regression analyses are presented in Table 3. When controlling for belief in dualism and determinism, greater belief in free will significantly predicted greater engagement in health protective behaviors and lesser engagement in health risk behaviors. The amount of variance explained ranged from 2% (for unhealthy snacking) to 35% (for physical activity).

**Table 3.** Study 2. Hierarchical Regression Analyses Predicting the Six Health Behaviors (*N* = 249).

Independent Variable	Physical Activity	Fruit and Vegetable	Low-Fat Diet	Alcohol Consumption	Smoking	Unhealthy Snacking
Step 1						
Determinism	−.050	−.097	−.089	.043	.054	.112
Dualism	.032	−.007	−.054	−.022	.075	.042
*R*^2^	.003	.010	.012	.002	.009	.015
*R*^2^_adj_	−.005	.002	.004	−.006	.001	.007
Step 2						
Determinism	−.055	−.100	−.093	.045	.056	.113
Dualism	.045	.002	−.044	−.030	.068	.039
Free will	.592***	.413***	.457***	−.345***	−.321***	−.146*
*R*^2^	.353	.180	.221	.121	.113	.037
*R*^2^_adj_	.345***	.170***	.211***	.110***	.102***	.025*
Δ*R*^2^	.350***	.170***	.209***	.119***	.103***	.021*

*Note.* *p < .05, ***p < .001.

### Discussion

Consistent with our expectations, Study 2 replicated the retrospective associations of Study 1 in a prospective context. Specifically, a significant positive relationship was found between free will belief measured at time 1 and health protective behaviors measured at time 2 and a significant negative relationship was found between free will belief measured at time 1 and health risk behaviors measured at time 2. Free will belief remained a significant and, in most cases, strong predictor of all health behaviors when controlling for belief in dualism and determinism. Indeed, effect sizes were large for physical activity; medium for fruit and vegetable consumption, low-fat diet, alcohol consumption, and smoking; and small for snacking (Cohen, 1992). The findings therefore suggest that believing in free will is associated with important health behaviors.

The two studies presented thus far established a relationship between belief in free will and past (Study 1) and prospective (Study 2) health behavior. As was previously mentioned, belief in free will relates to whether an individual has agentic control over at least some of their behaviors (Baumeister, 2008). However, there are other conceptually related constructs associated with human agency. We focus on three here: self-efficacy, health locus of control (HLOC), and implicit person theory.

Self-efficacy refers to an individual’s belief in their capability to undertake certain behaviors necessary to produce specific performance outcomes (Bandura, 1977). HLOC is the extent to which an individual perceives health outcomes to be either a consequence of internal or external sources (Wallston et al., 1978). Implicit theories are the extent to which a person believes attributes are either fixed or malleable (Dweck et al., 1995). We would expect a person with an internal locus of control, strong self-efficacy, and implicit person theory to possess greater belief in free will. Despite these associations, belief in free will should still independently predict behavior beyond these associated constructs.

## Study 3

### Purpose

The purpose of Study 3 was to examine belief in free will as a predictor of health behavior while controlling for HLOC, self-efficacy, and implicit theories.

### Method

#### Procedure

The study adopted the same design and procedure to that of Study 2. The study was pre-registered on AsPredicted (Ref: 146,903).

#### Measures

##### Time 1

Demographics of age and gender were taken. Belief in free will, determinism, and dualism were again assessed using the FWI (Nadelhoffer et al., 2014). HLOC was measured using 18 items (Wallston et al., 1978), with six items measuring internal HLOC (e.g., “If I get sick, it is my own behavior which determines how soon I get well again”, *α* = .78), six items measuring chance HLOC (e.g., “No matter what I do, if I am going to get sick, I will get sick”, *α* = .81), and six items measuring powerful others HLOC (e.g., “Having regular contact with my physician is the best way for me to avoid illness”, *α* = .82). All items used 6-point rating scales scored 1 (strongly disagree) to 6 (strongly agree). General self-efficacy was measured using 10 items (e.g., “I can always manage to solve difficult problems if I try hard enough”, *α* = .79) (Schwarzer & Jerusalem, 1995). These items used 4-point rating scales scored 1 (not at all true) to 4 (exactly true). Finally, implicit person theory was measured using three items (e.g., “The kind of person someone is, is something very basic about them and it can’t be changed very much”, *α* = .82) (Dweck et al., 1995). These items were scored 1 (strongly disagree) to 6 (strongly agree) using 6-point rating scales.

##### Time 2

To measure the six health behaviors, the six items used in studies 1 and 2 were again adopted.

#### Analyses

Correlations were computed between the FWI subscales, HLOC subscales, general self-efficacy, implicit person theory, and the six health behaviors. Following this, a series of hierarchical multiple regression analyses was undertaken to understand the predictive utility of free will beliefs on the health behaviors when controlling for HLOC, general self-efficacy, and implicit person theory. At step 1, HLOC, general self-efficacy, and implicit person theory were the independent variables, and the health behavior was the dependent variable. Belief in free will was then entered at Step 2.

### Results

A total of 287 participants completed T1 measures (186 males, 101 females; *M*_*age*_ = 21.84, *SD* = 2.69), with 262 participants providing responses at T2 (172 males, 90 females; *M*_*age*_ = 21.68, *SD* = 2.73). No differences were observed between participants completing both assessments and participants completing T1 measures only. The findings showed belief in free will exerted significant positive correlations with internal HLOC (*r* = .311, *p* < .001) and general self-efficacy (*r* = .143, *p* < .05). Thus, a person believing in free will is more likely to also possess greater internal HLOC and general self-efficacy. There was no association between belief in free will and implicit person theory. Like study 2, we found belief in free will positively correlated with fruit and vegetable consumption (*r* = .435, *p* < .001), physical activity (*r* = .528, *p* < .001), and low-fat diet (*r* = .428, *p* < .001), and negatively correlated with alcohol consumption (*r* = −.317, *p* < .001), unhealthy snacking (*r* = −.146, *p* < .05), and smoking (*r* = −.263, *p* < .001). Neither HLOC nor implicit person theory correlated with the health behaviors (all *p*s > .05). However, general self-efficacy did correlate with fruit and vegetable consumption (*r* = .199, *p* < .01), physical activity (*r* = .281, *p* < .001), and low-fat diet (*r* = .141, *p* < .05), indicating general self-efficacy beliefs are associated with positive health behaviors. All correlations can be seen in Supplementary File 1.

The regression analyses showed that at Step 1, the models significantly predicted physical activity, *F* (5, 256) = 5.823, *p* < .001, *R*^*2*^ = .102, and fruit and vegetable consumption, *F* (5, 256) = 3.072, *p* = .010, *R*^*2*^ = .057. General self-efficacy was the only significant predictor of these behaviors (physical activity: β = .264, *p* < .001; fruit and vegetable consumption: β = .199, *p* = .001). The inclusion of belief in free will at Step 2 significantly increased the explained variance in all six behaviors, and the models significantly predicted physical activity, *F* (6, 255) = 22.283, *p* < .001, *R*^*2*^ = .344, fruit and vegetable consumption, *F* (6, 255) = 13.085, *p* < .001, *R*^*2*^ = .235, low-fat diet, *F* (6, 255) = 12.092, *p* < .001, *R*^*2*^ = .222, alcohol consumption, *F* (6, 255) = 6.145, *p* < .001, *R*^*2*^ = .126, and smoking, *F* (6, 255) = 15.848, *p* < .01, *R*^*2*^ = .079. Across all six behaviors, belief in free will was the strongest predictor. Full results of regression analyses are presented in Table 4.

**Table 4.** Study 3. Hierarchical Regression Analyses Predicting the Six Health Behaviors (*N* = 262).

Independent Variable	Physical Activity	Fruit and Vegetable	Low-Fat Diet	Alcohol Consumption	Smoking	Unhealthy Snacking
Step 1						
HLOC internal	.089	−.015	−.038	.029	−.066	.012
HLOC chance	.059	−.119	−.081	.063	.124*	.043
HLOC others	.081	−.041	.017	.045	.031	.074
General self-efficacy	.264***	.199**	.140*	−.052	−.040	−.095
Implicit person theory	−.051	−.056	−.056	.119	.032	.062
*R*^2^	.102***	.057*	.039	.021	.023	.019
*R*^2^_adj_	.085***	.038*	.020	.002	.004	.000
Step 2						
HLOC internal	−.064	−.147*	−.071	.130*	.007	.055
HLOC chance	.013	−.073	−.079	.028	.098	.028
HLOC others	.076	−.045	.013	.048	.033	.075
General self-efficacy	.204***	.148**	.088	−.013	−.011	−.078
Implicit person theory	−.009	−.020	−.020	.092	.012	.050
Free will	.526***	.452***	.457***	−.347***	−.253***	−.148*
*R*^2^	.344***	.235***	.222***	.126***	.079**	.038
*R*^2^_adj_	.329***	.217***	.203***	.106***	.058**	.015
Δ*R*^2^	.242***	.179***	.183***	.105***	.056***	.019*

*Note.* *p < .05, **p < .01, ***p < .001.

### Discussion

The study again found associations between belief in free will and health behaviors. Specifically, belief in free will positively correlated with health protective behaviors and negatively correlated with health risk behaviors. More importantly, when controlling for HLOC, general self-efficacy, and implicit person theory, belief in free will remained a significant predictor of the health behaviors, and effect sizes were similar to those identified in studies 1 and 2. Moreover, belief in free will was with a better predictor of the health behaviors than the remaining constructs. Thus, locus of control, self-efficacy, and implicit theories do not account for the effects observed regarding free will beliefs. Belief in free will therefore appears to be separate to these conceptually associated constructs, and to predict health behaviors independent of such constructs.

Despite these findings, some of the conceptually associated constructs are rather generic. It could be that the effects of belief in free will on health behavior would be captured by more specific health-related cognitions. Study 4 advanced the findings of studies 1-3 by examining whether the social cognitive determinants outlined in the TPB are associated with and account for the effects of free will beliefs and health behavior.

## Study 4

We previously outlined that the TPB has been shown to explain health behavior (e.g., Armitage & Conner, 2001; Cooke & French, 2008; McEachan et al., 2011; Steinmetz et al., 2016). Moreover, in addition to the determinants outlined in the theory, the TPB considers the role of background factors in influencing behavior. However, rather than having a direct influence on behavior, theory determinants are theorized to mediate these additional factors. We suggested that attitude, subjective norm, PBC, and intention could mediate the relationship between free will beliefs and health behaviors, with these effects differing depending on whether the behavior is either health protective or health risk. Individuals believing more strongly in free will could have more positive attitudes and perceptions of control towards health protective behaviors and more negative attitudes and perceptions of control towards health positive behaviors. It has been shown that individuals possessing a greater belief in free will engender a greater sense of personal responsibility (Carey & Paulhus, 2013; Clark et al., 2017; Martin et al., 2017; Shariff et al., 2014). This sense of responsibility may shape attitudes towards people’s beneficial and detrimental health behaviors. Moreover, Crescioni et al. (2016) found a positive relationship between free will belief and self-efficacy, and St Quinton et al. (2022) found belief in free will was positively associated with attitude and control towards gambling behavior. Finally, research has shown that people believing more strongly in free will are more autonomous and less likely to conform (Alquist et al., 2013). Thus, free will beliefs could also play a role in how others are perceived in reference to health behaviors.

### Purpose

Advancing previous findings, the purpose of Study 4 was to understand the relationship between free will beliefs and TPB constructs in relation to health behavior. Given that physical activity and alcohol consumption demonstrated the strongest associations with free will beliefs from the respective health protective and health risk behaviors, these two behaviors were the focus of the study. It was that hypothesized greater belief in free will would be positively associated with intention to engage in health protective behaviors and negatively associated with intention to engage in health risk behaviors (H1). Greater free will beliefs were expected to be positively associated with attitude toward health protective behaviors and negatively associated with attitude toward health risk behaviors (H2); to be positively associated with subjective norm for health protective behaviors and negatively associated with subjective norm for health risk behaviors (H3); and to be positively associated with PBC for both health protective and health risk behaviors (H4). In accordance with the TPB, it was expected that attitude (H5), subjective norm (H6), and PBC (H7) would positively predict intention to engage in the health protective behaviors and negatively predict intention to engage in the health risk behaviors. Attitude, subjective norm, and PBC were expected to mediate the relationship between free will beliefs and intentions (H8-H10).

### Method

#### Procedure

A cross-sectional design was used. A similar procedure was followed to that of Study 1.

#### Measures

We again used subscales from the FWI (Nadelhoffer et al., 2014) to assess belief in free will. Additionally, attitude toward the two health behaviors were assessed using three items (e.g., “For me, participating in physical activity/alcohol consumption would be”, Unenjoyable-Enjoyable, *α* = .87 for physical activity and .81 for alcohol consumption); subjective norm was assessed using three items (e.g., “People who are important to me think that I should/should not engage in physical activity/alcohol consumption”, Should-Should not, *α* = .90 for physical activity and .84 for alcohol consumption); PBC was assessed using four items (e.g., “Whether or not I engage in physical activity/alcohol consumption is under my control”, False-True, *α* = .77 for physical activity and .75 for alcohol consumption); and intention was assessed using three items (e.g., “I plan to take part in physical activity/alcohol consumption”, Strongly agree-Strongly disagree, *α* = .95 for physical activity and .91 for alcohol consumption). These items were developed following the guidelines of Ajzen (2006) and previous studies (e.g., Cooke et al., 2016; Cooke & French, 2011). All items assessing social cognitive constructs adopted 7-point scales which varied in direction.

#### Analyses

To test the theorized direct and mediating relationships, Hayes’ PROCESS macro (Hayes, 2017) was used. Specifically, parallel mediation (Model 4) was undertaken bootstrapped with 5000 resamples and bias-corrected 95% confidence intervals.

### Results

Data were collected on 364 participants (210 males, 154 females; *M*_*age*_ = 22.84, *SD* = 3.36).

#### Physical activity

Results showed free will beliefs had a significant total effect on intention (*B* = 0.400, SE = 0.039, *p* < .001) and significant direct effects on attitude (*B* = 0.482, SE = 0.040, *p* < .001), subjective norm (*B* = 0.225, SE = 0.044, *p* < .001), and PBC (*B* = 0.529, SE = 0.039, *p* < .001). Attitude (*B* = 0.549, SE = 0.045, *p* < .001) and PBC (*B* = 0.143, SE = 0.046, *p* < .01) significantly predicted intention but subjective norm did not (*B* = −0.013, SE = 0.035, *p* > .05). This suggested the social cognitive constructs were candidate mediators for the free will belief-intention relationship. Mediation showed the indirect effects for attitude (*B* = 0.265, SE = 0.036, 95% CI = 0.198, 0.337) and PBC (*B* = 0.075, SE = 0.030, 95% CI = 0.018, 0.139) were significant. Free will beliefs did not predict intention when controlling for attitude, subjective norm, and PBC (B = 0.062, SE = 0.039, 95% CI = −0.014, 0.139), thus suggesting full mediation by attitude and PBC. These analyses are presented in Table 5.

**Table 5.** Study 4. Standardized and Unstandardized Parameter Estimates Predicting Physical Activity Intentions From Free Will Beliefs and Social Cognitive Constructs (*N* = 364).

Effect	*B*	SE	95% CI		Standardized *β*
			LB	UB	
Direct effects					
FW → Att	0.482***	0.040	.403	.562	0.531
FW → SN	0.225***	0.044	.138	.312	0.259
FW → PBC	0.529***	0.039	.451	.607	0.575
Att → Int	0.549***	0.045	.460	.638	0.588
SN → Int	−0.013	0.035	−.084	.056	−0.014
PBC → Int	0.143**	0.046	.051	.234	0.155
FW → Int	0.062	0.039	−.014	.139	0.073
Indirect effects					
FW → Att → Int	0.265	0.036	.198	.337	0.312
FW → SN → Int	−0.003	0.008	−.020	.012	−0.003
FW → PBC → Int	0.075	0.030	.018	.139	0.089
Total effect^a^					
FW → Int	0.400***	0.039	.323	.477	.471

*Note.* **p < .01, ***p < .001; ^a^Total effect comprising sums of all indirect effects through model constructs plus the direct effect; *B* = unstandardized parameter estimate; *β* = standardized parameter estimate; SE = standard error; 95% CI = 95% confidence interval of unstandardized parameter estimate; LB = lower bound of 95% CI; UB = upper bound of 95% CI; Att = attitude; FW = free will beliefs; Int = intention; PBC = perceived behavioral control; SN = subjective norm.

#### Alcohol consumption

Results showed free will beliefs had a significant total effect on intention (*B* = −0.246, SE = 0.043, *p* < .001) and significant direct effects on attitude (*B* = −0.271, SE = 0.044, *p* < .001), subjective norm (*B* = −0.107, SE = 0.039, *p* < .01), and PBC (*B* = 0.424, SE = 0.043, *p* < .001). Attitude (*B* = 0.605, SE = 0.036, *p* < .001), subjective norm (*B* = 0.215, SE = 0.040, *p* < .001), and PBC (*B* = −0.118, SE = 0.036, *p* < .01) significantly predicted intention. This suggested the social cognitive constructs were candidate mediators for the free will belief-intention relationship. Mediation showed the indirect effects for attitude (*B* = −0.164, SE = 0.030, 95% CI = −0.222, −0.108), subjective norm (*B* = 0.023, SE = 0.010, 95% CI = −0.046, −0.004), and PBC (*B* = −0.050, SE = 0.015, 95% CI = −0.080, −0.021) were significant. Free will beliefs did not predict intention when controlling for attitude, subjective norm, and PBC (B = −0.008, SE = 0.034, 95% CI = −0.076, 0.059), thus suggesting full mediation by these constructs. These analyses are presented in Table 6.

**Table 6.** Study 4. Standardized and Unstandardized Parameter Estimates Predicting Alcohol Consumption Intentions From Free Will Beliefs and Social Cognitive Constructs (*N* = 364).

Effect	*B*	SE	95% CI		Standardized *β*
			LB	UB	
Direct effects					
FW → Att	−0.271***	0.044	−0.359	−0.182	−0.302
FW → SN	−0.107**	0.039	−0.184	−0.029	−0.140
FW → PBC	0.424***	0.043	0.338	0.510	0.456
Att → Int	0.605***	0.036	0.534	0.676	0.634
SN → Int	0.215***	0.040	0.135	0.295	0.191
PBC → Int	−0.118**	0.036	−0.189	−0.046	−0.128
FW → Int	−0.008	0.034	−0.076	0.059	−0.010
Indirect effects					
FW → Att → Int	−0.164	0.030	−0.222	−0.108	−0.192
FW → SN → Int	−0.023	0.010	−0.046	−0.004	−0.027
FW → PBC → Int	−0.050	0.015	−0.080	−0.021	−0.058
Total effect^a^					
FW → Int	−0.246***	0.043	−0.330	−0.161	−0.287

*Note.* **p < .01, ***p < .001; ^a^Total effect comprising sums of all indirect effects through model constructs plus the direct effect; *B* = unstandardized parameter estimate; *β* = standardized parameter estimate; SE = standard error; 95% CI = 95% confidence interval of unstandardized parameter estimate; LB = lower bound of 95% CI; UB = upper bound of 95% CI; Att = attitude; FW = free will beliefs; Int = intention; PBC = perceived behavioral control; SN = subjective norm.

### Discussion

Study 4 examined the relationship between free will beliefs and social cognitive constructs in relation to two health behaviors. As we predicted and in accordance with previous work (e.g., Cooke et al., 2016), intention to engage in alcohol consumption was predicted by attitude, subjective norm, and PBC. With regards to physical activity, attitude and PBC predicted intention, but subjective norm did not. Although only providing partial support for our hypotheses, these findings support previous studies demonstrating the importance of attitude and PBC in physical activity (e.g., Hamilton & White, 2008; Plotnikoff et al., 2013).

More pertinent to the study, and as predicted, results showed free will beliefs significantly predicted attitude, subjective norm, PBC, and intention towards both behaviors. Thus, believing in free will demonstrated significant associations with key social cognitive constructs. In relation to physical activity, the positive relations suggest those possessing a stronger belief in free will generally have more positive attitudes toward exercise, more perceived social support, and greater perceived control over the behavior. In relation to alcohol consumption, the negative relationship with attitude and subjective norm suggests those possessing a stronger belief in free will generally have less positive attitudes towards consuming alcohol and less perceived social approval towards the behavior. The positive relationship with PBC suggests that the stronger the belief in free will the stronger a person believes alcohol consumption is under their control. Taken together, these findings indicate that when it comes to the key psychological determinants underlying health behaviors, it is more beneficial to believe in free will.

Mediation analysis found significant indirect effects for free will beliefs on intention in both behaviors. Specifically, attitude and PBC mediated the relationship between free will belief and intention to engage in physical activity; and attitude, subjective norm, and PBC mediated the relationship between free will belief and intention to engage in alcohol consumption. These findings suggest that, to the extent that intentions towards the health behaviors are influenced by belief in free will, these influences occur via changes to the social cognitive constructs outlined in the TBP. This supports TPB assertions that free will beliefs operate as distal factors to more proximal theory determinants (Ajzen, 1991).

## General Discussion

Recent attention has been given to whether believing in free will has positive societal outcomes. Our results indicate that free will beliefs are associated with both greater health protective (e.g., physical activity) and reduced health risk (e.g., alcohol consumption) behaviors. Specifically, we found that stronger free will beliefs were positively associated with both past (Study 1) and prospective (Study 2 and 3) health promoting behaviors and negatively associated with both past (Study 1) and prospective (Study 2 and 3) health risk behaviors. We showed belief in free will to be distinct from conceptually related psychological constructs, and to predict health behaviors independent of such constructs (Study 3). Finally, we found constructs from the TPB fully mediated the relationship between free will belief and the intention to engage in two health behaviors, physical activity and alcohol consumption (Study 4). Taken together, the studies suggest that, as well as social behaviors such as cheating (Vohs & Schooler, 2008), prejudice (Zhao et al., 2014), and aggressiveness (Baumeister et al., 2009), free will beliefs are also associated with important health behaviors. Thus, the present findings further support previous research demonstrating that belief in free will is associated with health-related behaviors (e.g., St Quinton et al., 2022; Vonasch et al., 2017).

It is interesting that the health protective behaviors had stronger associations with belief in free will than did the health risk behaviors. It could be that believing in free will motivates people to engage in behaviors that are positive more so than a lack of free will demotivates people to overcome urges and impulses. Such an interpretation would be consistent with past findings that have linked greater belief in free will to setting more meaningful goals (Crescioni et al., 2016; Zhao et al., 2023), greater self-efficacy (Crescioni et al., 2016), and greater perseverance for long-term goals (Li et al., 2018). Nevertheless, we did find significant associations with the health risk behaviors, thus suggesting free will also plays a role in these detrimental health behaviors. It is also interesting that belief in free will was most strongly associated with physical activity. Characteristics associated with a belief in free will, such as autonomy, choice, and responsibility, have also been shown to be important in physical activity (Hagger et al., 2005; How et al., 2013). Thus, it is unsurprising that there was a strong positive relationship between physical activity and belief in free will.

The present findings suggest interventions that increase belief in free will could have positive implications for health behaviors. Strengthening free will beliefs could lead to greater participation in health protecting behaviors and less participation in health risk behaviors. Conversely, attenuating free will beliefs could lead to less participation in health protecting behaviors and greater participation in health risk behaviors. Although some studies have observed a number of behavioral consequences from successful free will belief manipulations (e.g., Alquist et al., 2013; Crescioni et al., 2016; Vohs & Schooler, 2008; Zhao et al., 2014), it should be noted that one should be conservative in their expectations about behavioral change from such manipulations. Indeed, a recent meta-analysis on free will belief manipulation studies found successful manipulations had only small effects on behavior (Genschow et al., 2023). Yet, this should come as no surprise as large changes in beliefs often lead to small effects on behavior (Sniehotta et al., 2014; Webb & Sheeran, 2006). Future research should therefore examine the extent to which free will belief manipulations lead to changes in health behaviors.

When these manipulations are undertaken, researchers should also take measures of constructs outlined in the TPB. Our results suggest specific determinants would mediate successful manipulation attempts. For example, in relation to physical activity it would be expected that belief in free will would have indirect effects on behavior through attitude and PBC. And in relation to alcohol consumption, we would expect attitude, subjective norm, and PBC to mediate between beliefs in free will and the behavior. Research confirming these associations would provide important information about the underlying mechanisms through which free will beliefs exert their effects on health behavior. Thus, belief in free will may not only influence participation in health behaviors, but it may also broadly influence key social psychological determinants.

The present research provides preliminary evidence for the association between free will beliefs and health behavior. However, these studies were not without limitations. First, like the many correlational studies investigating belief in free will, no causal conclusions can be made about the relationship between free will belief and health behaviors. Free will beliefs may influence health behavior or health behavior may influence free will beliefs; for example, people who generally engage in health protective behaviors may be inclined to view themselves as highly agentic and responsible for their good health, whereas those who frequently engage in health risk behaviors may be inclined to disavow agency as a means of excusing those behaviors. Research should establish the causal role of free will beliefs on health behavior through experimental or longitudinal work. Second, the reliance on self-report could have led to bias. We would encourage replications of our findings using more direct observations of health behaviors, especially due to replication difficulties in this area (Crone & Levy, 2019; Nadelhoffer et al., 2020; Shariff & Vohs, 2014; St Quinton et al., 2023). However, it is worth noting that the patterns across all four studies were consistent. Third, the recruitment strategy likely meant the research was conducted on Western samples. Research has shown belief in free will potentially has a cultural component (e.g., Berniūnas et al., 2021; Feldman et al., 2018). It may therefore be useful for research to examine the relationship between belief in free will and health behavior in different cultural contexts. Finally, we investigated only a limited number of health behaviors and used a single social cognitive theory to explain potential mechanisms. Future research should therefore examine the role of free will beliefs in a broader range of health behaviors, whilst also considering additional theories and potential explanatory pathways (St Quinton & Crescioni, 2022).

## Conclusion

Belief in free will has demonstrated a role in many social behaviors. The present research suggests that regardless of whether free will actually exists, believing it does motivates people to engage in healthier behaviors. Moreover, the relationship between belief in free will and health behavior appear to be associated with key social cognitive constructs. Although future research is needed to causally validate these relations, it appears that in terms of health behaviors, it is more beneficial for people to believe in free will than to not hold these views.

## Supplemental Material

Supplemental Material - Free to be Healthy? Free Will Beliefs are Positively Associated With Health Behavior Supplemental Material for Free to be Healthy? Free Will Beliefs are Positively Associated With Health Behavior by Tom St Quinton, and A. William Crescioni in Psychological Reports.

## Data Availability

The data that support the findings of this article are available on request from the corresponding author, TSQ.

## References

[bibr1-00332941241260264] Ajzen I. (1991). The theory of planned behavior. Organizational Behavior and Human Decision Processes, 50(2), 179–211. 10.1016/0749-5978(91)90020-T

[bibr2-00332941241260264] Ajzen I. (2006). Constructing a TPB questionnaire: Conceptual and methodological considerations. https://www.people.umass.edu/aizen/pdf/tpb.measurement.pdf

[bibr3-00332941241260264] Ajzen I. (2011). The theory of planned behaviour: Reactions and reflections. Psychology and Health, 26(9), 1113–1127. 10.1080/08870446.2011.613995 21929476

[bibr4-00332941241260264] Alquist J. L. Ainsworth S. E. Baumeister R. F. (2013). Determined to conform: Disbelief in free will increases conformity. Journal of Experimental Social Psychology, 49(1), 80–86. 10.1016/j.jesp.2012.08.015

[bibr5-00332941241260264] Alquist J. L. Ainsworth S. E. Baumeister R. F. Daly M. Stillman T. F. (2015). The making of might-have-beens: Effects of free will belief on counterfactual thinking. Personality and Social Psychology Bulletin, 41(2), 268–283. 10.1177/0146167214563673 25511569

[bibr6-00332941241260264] Armitage C. J. Conner M. (2001). Efficacy of the theory of planned behaviour: A metaEfficacy of the theory of planned behaviour: A meta-analytic reviewanalytic review. British Journal of Social Psychology, 40(Pt 4), 471–499. 10.1348/014466601164939 11795063

[bibr7-00332941241260264] Bandura A. (1977). Self-efficacy: Toward a unifying theory of behavioral change. Psychological Review, 84(2), 191–215. 10.1037/0033-295X.84.2.191 847061

[bibr8-00332941241260264] Baumeister R. F. (2008). Free will in scientific psychology. Perspectives on Psychological Science: A Journal of the Association for Psychological Science, 3(1), 14–19. 10.1111/j.1745-6916.2008.00057.x 26158665

[bibr9-00332941241260264] Baumeister R. F. Masicampo E. J. DeWall C. N. (2009). Prosocial benefits of feeling free: Disbelief in free will increases aggression and reduces helpfulness. Personality and Social Psychology Bulletin, 35(2), 260–268. 10.1177/0146167208327217 19141628

[bibr10-00332941241260264] Baumeister R. F. Sparks E. A. Stillman T. F. Vohs K. D. (2008). Free will in consumer behavior: Self-control, ego depletion, and choice. Journal of Consumer Psychology, 18(1), 4–13. 10.1016/j.jcps.2007.10.002

[bibr11-00332941241260264] Berniūnas R. Beinorius A. Dranseika V. Silius V. Rimkevičius P. (2021). The weirdness of belief in free will. Consciousness and Cognition, 87, Article 103054. 10.1016/j.concog.2020.103054 33254053

[bibr12-00332941241260264] Carey J. M. Paulhus D. L. (2013). Worldview implications of believing in free will and/or determinism: Politics, morality, and punitiveness. Journal of Personality, 81(2), 130–141. 10.1111/j.1467-6494.2012.00799.x 22812486

[bibr13-00332941241260264] Clark C. J. Baumeister R. F. Ditto P. H. (2017). Making punishment palatable: Belief in free will alleviates punitive distress. Consciousness and Cognition, 51, 193–211. 10.1016/j.concog.2017.03.010 28388484

[bibr14-00332941241260264] Cohen J. (1992). Statistical power analysis. Current Directions in Psychological Science, 1(3), 98–101. 10.1111/1467-8721.ep10768783

[bibr15-00332941241260264] Conner M. Abraham C. (2001). Conscientiousness and the theory of planned behavior: Toward a more complete model of the antecedents of intentions and behavior. Personality and Social Psychology Bulletin, 27(11), 1547–1561. 10.1177/01461672012711014

[bibr16-00332941241260264] Conner M. Wilding S. Prestwich A. Hutter R. Hurling R. Harreveld F. v. Abraham C. Sheeran P. (2022). Goal prioritization and behavior change: Evaluation of an intervention for multiple health behaviors. Health Psychology: Official Journal of the Division of Health Psychology, American Psychological Association, 41(5), 356–365. 10.1037/hea0001149 35467903

[bibr17-00332941241260264] Cooke R. Dahdah M. Norman P. French D. P. (2016). How well does the theory of planned behaviour predict alcohol consumption? A systematic review and meta-analysis. Health Psychology Review, 10(2), 148–167. 10.1080/17437199.2014.947547 25089611 PMC4867851

[bibr18-00332941241260264] Cooke R. French D. P. (2008). How well do the theory of reasoned action and theory of planned behaviour predict intentions and attendance at screening programmes? A meta-analysis. Psychology and Health, 23(7), 745–765. 10.1080/08870440701544437 25160879

[bibr19-00332941241260264] Cooke R. French D. P. (2011). The role of context and timeframe in moderating relationships within the theory of planned behaviour. Psychology and Health, 26(9), 1225–1240. 10.1080/08870446.2011.572260 21678186

[bibr20-00332941241260264] Crescioni A. W. Baumeister R. F. Ainsworth S. E. Ent M. Lambert N. M. (2016). Subjective correlates and consequences of belief in free will. Philosophical Psychology, 29(1), 41–63. 10.1080/09515089.2014.996285

[bibr21-00332941241260264] Crone D. L. Levy N. L. (2019). Are free will believers nicer people? (Four studies suggest not). Social Psychological and Personality Science, 10(5), 612–619. 10.1177/1948550618780732 31249653 PMC6542011

[bibr22-00332941241260264] de Leeuw A. Valois P. Ajzen I. Schmidt P. (2015). Using the theory of planned behavior to identify key beliefs underlying pro-environmental behavior in high-school students: Implications for educational interventions. Journal of Environmental Psychology, 42(8), 128–138. 10.1016/j.jenvp.2015.03.005

[bibr23-00332941241260264] Dweck C. S. Chiu C. Y. Hong Y. Y. (1995). Implicit theories and their role in judgments and reactions: A word from two perspectives. Psychological Inquiry, 6(4), 267–285. 10.1207/s15327965pli0604_1

[bibr24-00332941241260264] Feldman G. Farh J. L. Wong K. F. E. (2018). Agency beliefs over time and across cultures: Free will beliefs predict higher job satisfaction. Personality and Social Psychology Bulletin, 44(3), 304–317. 10.1177/0146167217739261 29191084 PMC5810915

[bibr25-00332941241260264] Genschow O. Cracco E. Schneider J. Protzko J. Wisniewski D. Brass M. Schooler J. W. (2023). Manipulating belief in free will and its downstream consequences: A meta-analysis. Personality and Social Psychology Review: An Official Journal of the Society for Personality and Social Psychology, Inc, 27(1), 52–82. 10.1177/10888683221087527 35676864

[bibr26-00332941241260264] Hagger M. S. Chatzisarantis N. L. D. Barkoukis V. Wang C. K. J. Baranowski J. (2005). Perceived autonomy support in physical education and leisure-time physical activity: A cross-cultural evaluation of the trans-contextual model. Journal of Educational Psychology, 97(3), 376–390. 10.1037/0022-0663.97.3.376

[bibr27-00332941241260264] Hagger M. S. Hamilton K. (2022). Predicting COVID-19 booster vaccine intentions. Applied psychology. Health and well-being, 14(3), 819–841. 10.1111/aphw.12349 35193171 PMC9111247

[bibr28-00332941241260264] Hamilton K. White K. M. (2008). Extending the theory of planned behavior: The role of self and social influences in predicting adolescent regular moderate-to-vigorous physical activity. Journal of Sport & Exercise Psychology, 30(1), 56–74. 10.1123/jsep.30.1.56 18369243

[bibr29-00332941241260264] Hayes A. F. (2017). Introduction to mediation, moderation, and conditional process analysis: A regression-based approach. Guilford Publications.

[bibr30-00332941241260264] How Y. M. Whipp P. Dimmock J. Jackson B. (2013). The effects of choice on autonomous motivation, perceived autonomy support, and physical activity levels in high school physical education. Journal of Teaching in Physical Education, 32(2), 131–148. 10.1123/jtpe.32.2.131

[bibr31-00332941241260264] Li J. Zhao Y. Lin L. Chen J. Wang S. (2018). The freedom to persist: Belief in free will predicts perseverance for long-term goals among Chinese adolescents. Personality and Individual Differences, 121(15), 7–10. 10.1016/j.paid.2017.09.011

[bibr32-00332941241260264] Martin N. D. Rigoni D. Vohs K. D. (2017). Free will beliefs predict attitudes toward unethical behavior and criminal punishment. Proceedings of the National Academy of Sciences of the United States of America, 114(28), 7325–7330. 10.1073/pnas.1702119114 28652361 PMC5514725

[bibr33-00332941241260264] McEachan R. R. C. Conner M. Taylor N. J. Lawton R. J. (2011). Prospective prediction of health-related behaviours with the theory of planned behaviour: A meta-analysis. Health Psychology Review, 5(2), 97–144. 10.1080/17437199.2010.521684

[bibr34-00332941241260264] McEachan R. R. C. Sutton S. Myers L. (2010). Mediation of personality influences on physical activity within the theory of planned behaviour. Journal of Health Psychology, 15(8), 1170–1180. 10.1177/1359105310364172 20522504

[bibr35-00332941241260264] Moynihan A. B. Igou E. R. van Tilburg W. A. (2019). Lost in the crowd: Conformity as escape following disbelief in free will. European Journal of Social Psychology, 49(3), 503–520. 10.1002/ejsp.2499

[bibr36-00332941241260264] Nadelhoffer T. Shepard J. Crone D. L. Everett J. A. Earp B. D. Levy N. (2020). Does encouraging a belief in determinism increase cheating? Reconsidering the value of believing in free will. Cognition, 203, Article 104342. 10.1016/j.cognition.2020.104342 32593841

[bibr37-00332941241260264] Nadelhoffer T. Shepard J. Nahmias E. Sripada C. Ross L. T. (2014). The free will inventory: Measuring beliefs about agency and responsibility. Consciousness and Cognition, 25(1), 27–41. 10.1016/j.concog.2014.01.006 24561311

[bibr38-00332941241260264] Ntoumanis N. Ng J. Y. Prestwich A. Quested E. Hancox J. E. Thøgersen-Ntoumani C. Deci E. L. Ryan R. M. Lonsdale C. Williams G. C. (2021). A meta-analysis of self-determination theory-informed intervention studies in the health domain: Effects on motivation, health behavior, physical, and psychological health. Health Psychology Review, 15(2), 214–244. 10.1080/17437199.2020.1718529 31983293

[bibr39-00332941241260264] Plotnikoff R. C. Costigan S. A. Karunamuni N. Lubans D. R. (2013). Social cognitive theories used to explain physical activity behavior in adolescents: A systematic review and meta-analysis. Preventive Medicine, 56(5), 245–253. 10.1016/j.ypmed.2013.01.013 23370047

[bibr40-00332941241260264] Rigoni D. Kühn S. Gaudino G. Sartori G. Brass M. (2012). Reducing self-control by weakening belief in free will. Consciousness and Cognition, 21(3), 1482–1490. 10.1016/j.concog.2012.04.004 22579497

[bibr41-00332941241260264] Schönbrodt F. D. Perugini M. (2013). At what sample size do correlations stabilize? Journal of Research in Personality, 47(5), 609–612. 10.1016/j.jrp.2013.05.009

[bibr42-00332941241260264] Schüz B. Conner M. Wilding S. Alhawtan R. Prestwich A. Norman P. (2021). Do socio-structural factors moderate the effects of health cognitions on COVID-19 protection behaviours? Social Science & Medicine, 285, Article 114261. 10.1016/j.socscimed.2021.114261 PMC8299154 34332252

[bibr43-00332941241260264] Schwarzer R. Jerusalem M. (1995). Generalized self-efficacy scale. In Weinman J. Wright S. Johnston M. (Eds.), Measures in health psychology: A user’s portfolio. Causal and control beliefs (pp. 35–37). NFER-NELSON.

[bibr44-00332941241260264] Shariff A. F. Greene J. D. Karremans J. C. Luguri J. B. Clark C. J. Schooler J. W. Baumeister R. F. Vohs K. D. (2014). Free will and punishment: A mechanistic view of human nature reduces retribution. Psychological Science, 25(8), 1563–1570. 10.1177/0956797614534693 24916083

[bibr45-00332941241260264] Shariff A. F. Vohs K. D. (2014). The world without free will. Scientific American, 310(6), 76–79. 10.1038/scientificamerican0614-76 25004579

[bibr46-00332941241260264] Sheeran P. Conner M. (2019). Degree of reasoned action predicts increased intentional control and reduced habitual control over health behaviors. Social Science & Medicine, 228(7), 68–74. 10.1016/j.socscimed.2019.03.015 30884424

[bibr47-00332941241260264] Sniehotta F. F. Presseau J. Araújo-Soares V. (2014). Time to retire the theory of planned behaviour. Health Psychology Review, 8(1), 1–7. 10.1080/17437199.2013.869710 25053004

[bibr48-00332941241260264] Steinmetz H. Knappstein M. Ajzen I. Schmidt P. Kabst R. (2016). How effective are behavior change interventions based on the theory of planned behavior? Zeitschrift für Psychologie, 224(3), 216–233. 10.1027/2151-2604/a000255

[bibr49-00332941241260264] St Quinton T. Crescioni A. W. (2022). Belief in free will: Integration into social cognition models to promote health behavior. Philosophical Psychology, 1–19. 10.1080/09515089.2022.2140649

[bibr50-00332941241260264] St Quinton T. Morris B. Crescioni A. W. (2022). Beliefs in free will and determinism: Associations with social cognition and gambling behavior. Addiction Research and Theory, 30(6), 414–421. 10.1080/16066359.2022.2062330

[bibr51-00332941241260264] St Quinton T. Trafimow D. Genschow O. (2023). The role of free will beliefs in social behavior: Priority areas for future research. Consciousness and Cognition, 115(1), Article 103586. 10.1016/j.concog.2023.103586 37837797

[bibr52-00332941241260264] Van Inwagen P. (2008). How to think about the problem of free will. The Journal of Ethics, 12(3-4), 327–341. 10.1007/s10892-008-9038-7

[bibr53-00332941241260264] Vohs K. D. Schooler J. W. (2008). The value of believing in free will: Encouraging a belief in determinism increases cheating. Psychological Science, 19(1), 49–54. 10.1111/j.1467-9280.2008.02045.x 18181791

[bibr54-00332941241260264] Vonasch A. J. Clark C. J. Lau S. Vohs K. D. Baumeister R. F. (2017). Ordinary people associate addiction with loss of free will. Addictive Behaviors Reports, 5, 56–66. 10.1016/j.abrep.2017.01.002 29450228 PMC5800573

[bibr55-00332941241260264] Wallston K. A. Wallston B. S. DeVellis R. (1978). Development of the multidimensional health locus of control (MHLC) scales. Health Education Monographs, 6(2), 160–170. 10.1177/109019817800600107 689890

[bibr56-00332941241260264] Webb T. L. Sheeran P. (2006). Does changing behavioral intentions engender behavior change? A meta-analysis of the experimental evidence. Psychological Bulletin, 132(2), 249–268. https://psycnet.apa.org/doi/10.1037/0033-2909.132.2.249 16536643 10.1037/0033-2909.132.2.249

[bibr57-00332941241260264] Will Crescioni A. Ehrlinger J. Alquist J. L. Conlon K. E. Baumeister R. F. Schatschneider C. Dutton G. R. (2011). High trait self-control predicts positive health behaviors and success in weight loss. Journal of Health Psychology, 16(5), 750–759. 10.1177/1359105310390247 21421645 PMC4675362

[bibr58-00332941241260264] Zhao M. Liu J. Huo Y. (2023). The value of believing in free will: A prediction on seeking and experiencing meaning in life. Applied Psychology: Health and WellApplied psychology. Health and well-beingBeing, 16(2), 537–552. 10.1111/aphw.12503 37848383

[bibr59-00332941241260264] Zhao X. Liu L. Zhang X.-x. Shi J.-x. Huang Z.-w. (2014). The effect of belief in free will on prejudice. PLoS One, 9(3), Article e91572. 10.1371/journal.pone.0091572 24622280 PMC3951431

