# Supplementary material for: Free to be Healthy? Free Will Beliefs are Positively Associated With Health Behavior
Source: Psychol Rep. 2024 Jun 8;129(2):1404–28. doi: 10.1177/00332941241260264 (PMC12953652; doi:10.1177/00332941241260264)
Supplement: Supplemental Material - Free to be Healthy? Free Will Beliefs are Positively Associated With Health Behavior [file sj-pdf-1-prx-10.1177_00332941241260264.pdf]

Supplementary file 1.

Study 3. Means, standard deviations, and zero-order correlations (N = 262)

	M (SD)	1	2	3	4	5	6	7	8	9	10	11	12	13	14
1. Free will	4.02 (1.65)	--													
2. Determinism	3.01 (1.22)	-.002	--												
3. Dualism	3.23 (1.45)	-.031	.139*	--											
4. Implicit person theory	3.83 (1.60)	-.096	-.046	.009	--										
5. HLOC internal	3.73 (1.64)	.311***	-.039	-.004	-.053	--									
6. HLOC chance	3.38 (1.53)	-.108	-.110	-.022	-.099	-.056	--								
7. HLOC others	3.59 (1.64)	.056	.092	.085*	-.112	.105	-.027	--							
8. General self-efficacy	2.70 (1.01)	.143*	.108	.057	-.093	.079	.020	.050	--						
9. Physical activity	3.31 (1.56)	.528***	-.041	.052	-.095	.118	.063	.107	.281***	--					
10. Fruit and vegetable	3.67 (1.45)	.435***	-.087*	-.013	-.057	.006	-.108	-.023	.199**	.478***	--				
11. Low-fat diet	3.51 (2.01)	.428***	-.099	-.033	-.056	-.015	-.116	.029	.141*	.499***	.748***	--			
12. Alcohol consumption	3.74 (1.84)	-.317***	.051	-.028	.111	.020	.047	.031	-.057	-.254***	-.237***	-.228***	--		
13. Smoking	2.11 (2.01)	-.263***	.071	.072	.023	-.075	.123	.015	-.044	-.292***	-.301***	-.243***	.289***	--	
14. Unhealthy snacking	3.16 (2.05)	-.146*	.116	.034	.058	.007	.051	.063	-.096	-.227***	-.249***	-.215***	.221***	.539***	--

Note. *p < .05, **p < .01, ***p < .001
